# How to customize common data models for rare diseases: an OMOP-based implementation and lessons learned

**DOI:** 10.1186/s13023-024-03312-9

**Published:** 2024-08-14

**Authors:** Najia Ahmadi, Michele Zoch, Oya Guengoeze, Carlo Facchinello, Antonia Mondorf, Katharina Stratmann, Khader Musleh, Hans-Peter Erasmus, Jana Tchertov, Richard Gebler, Jannik Schaaf, Lena S. Frischen, Azadeh Nasirian, Jiabin Dai, Elisa Henke, Douglas Tremblay, Andrew Srisuwananukorn, Martin Bornhäuser, Christoph Röllig, Jan-Niklas Eckardt, Jan Moritz Middeke, Markus Wolfien, Martin Sedlmayr

**Affiliations:** 1 Institute for Medical Informatics and Biometry, Carl Gustav Carus Faculty of Medicine, TUD Dresden University of Technology, Fetscherstraße 74, 01307 Dresden, Germany; 2 Department of Internal Medicine I, University Hospital Frankfurt, Goethe University, Frankfurt, Germany; 3 Goethe University Frankfurt, University Hospital, Institute of Medical Informatics, Frankfurt, Germany; 4 University Hospital Frankfurt, Goethe University, Executive Department for Medical IT-Systems and Digitalization, Frankfurt, Germany; 5 Tisch Cancer Institute, Icahn School of Medicine at Mount Sinai, New York, NY USA; 6 Ohio State Comprehensive Cancer Center, Columbus, OH USA; 7 Center of Medical Informatics, University Hospital Carl Gustav Carus, TUD Dresden University of Technology, Dresden, Germany; 8 Department of Internal Medicine I, University Hospital Carl Gustav Carus, TUD Dresden University of Technology, Dresden, Germany; 9 Else-Kroener-Fresenius-Center for Digital Health, TUD Dresden University of Technology, Dresden, Germany; 10 Center for Scalable Data Analytics and Artificial Intelligence (ScaDS.AI) Dresden/Leipzig, Dresden, Germany

**Keywords:** Common data model, Rare disease, Interoperability, Data standardization, OMOP, OHDSI, Genotypes and phenotypes, Multi-center studies, Artificial intelligence

## Abstract

**Background:**

Given the geographical sparsity of Rare Diseases (RDs), assembling a cohort is often a challenging task. Common data models (CDM) can harmonize disparate sources of data that can be the basis of decision support systems and artificial intelligence-based studies, leading to new insights in the field. This work is sought to support the design of large-scale multi-center studies for rare diseases.

**Methods:**

In an interdisciplinary group, we derived a list of elements of RDs in three medical domains (endocrinology, gastroenterology, and pneumonology) according to specialist knowledge and clinical guidelines in an iterative process. We then defined a RDs data structure that matched all our data elements and built Extract, Transform, Load (ETL) processes to transfer the structure to a joint CDM. To ensure interoperability of our developed CDM and its subsequent usage for further RDs domains, we ultimately mapped it to Observational Medical Outcomes Partnership (OMOP) CDM. We then included a fourth domain, hematology, as a proof-of-concept and mapped an acute myeloid leukemia (AML) dataset to the developed CDM.

**Results:**

We have developed an OMOP-based rare diseases common data model (RD-CDM) using data elements from the three domains (endocrinology, gastroenterology, and pneumonology) and tested the CDM using data from the hematology domain. The total study cohort included 61,697 patients. After aligning our modules with those of Medical Informatics Initiative (MII) Core Dataset (CDS) modules, we leveraged its ETL process. This facilitated the seamless transfer of demographic information, diagnoses, procedures, laboratory results, and medication modules from our RD-CDM to the OMOP. For the phenotypes and genotypes, we developed a second ETL process. We finally derived lessons learned for customizing our RD-CDM for different RDs.

**Discussion:**

This work can serve as a blueprint for other domains as its modularized structure could be extended towards novel data types. An interdisciplinary group of stakeholders that are actively supporting the project's progress is necessary to reach a comprehensive CDM.

**Conclusion:**

The customized data structure related to our RD-CDM can be used to perform multi-center studies to test data-driven hypotheses on a larger scale and take advantage of the analytical tools offered by the OHDSI community.

**Supplementary Information:**

The online version contains supplementary material available at 10.1186/s13023-024-03312-9.

## Background

Rare diseases (RD) can be seen as an intricate puzzle in medical research, compelling us to decipher their complex phenotypes and genetic origins to unlock the potential for personalized therapeutic interventions. In the US, a disease is considered rare, if it affects fewer than 200,000 people [1], while in Europe, it is 5 per 10,000 people [2]. About 7,000 RD are known to affect 3.5–5.9% of the world’s population [2, 3]. About 80% of RD have a genetic basis [2–6], resulting from mutations in an individual's genome that can be inherited through parental chromosomes [6].

Timely and accurate identification of RD is oftentimes dependent on multiple factors, such as the amount and variety of individual patient information and comparable data, as well as knowledge about the specific RD itself [7]. The large variety and infrequent, highly individual nature of RD patients that physicians encounter create a major challenge in diagnosis and treatment [8]. Moreover, assembling a sufficiently large study cohort to investigate and characterize specific RD bears an additional challenge [9]. Here, one key aspect is the availability of disease-specific data, including genotype and phenotype information that can be used for the in-depth characterization and accurate identification of RD. Since diagnosis of RD, especially those with a genetic background, based solely on non-genetic clinical features is often misleading, or inaccurate [10], a genetic underpinning of the diagnosis is inevitable to allow identification of a more precise molecular cause that may explain the clinical phenotype [5]. However, due to genotype and phenotype variability, lack of knowledge about individual gene variations and their interplay, as well as an individual patient journey, the investigation of RD requires a more customized approach. This is also reflected in the defination of personalised medicine by the Horizon 2020 Advisory Group as “*a medical model using the characterization of individuals’ phenotypes and genotypes (e.g., molecular profiling, medical imaging, lifestyle data) for tailoring the right therapeutic strategy for the right person at the right time, and/or to determine the predisposition to disease and/or to deliver timely and targeted prevention.”* [11].

To allow for such an improved and extended investigation of RD, multi-center studies are an inevitable asset to cover a reasonably large patient cohort. However, these also increase the computational complexity. Here, novel computational concepts that satisfy FAIR (Findable, Accessible, Interoperable, and Reusable) Data Principles [12, 13] are necessary to obtain more transparent and sustainable diagnostic processes at a large scale [13, 14]. In addition, data from various study sites need to be in the same format, which can be attributed to the different layers of interoperability [15, 16]. Consequently, the syntactic and semantic interoperability of the source data of the study sites has to be ensured. Syntactic interoperability refers to the harmonization and definition of data formats, as well as information models for the specification of information units and their interface whereas semantic interoperability focuses on the enablement of shared understanding of message content between systems and/or users [17].

During the last decade, efforts have been made to reach syntactic and semantic interoperability in the medical domain. Communications standards, such as Fast Healthcare Interoperability Resources (FHIR) [18], are often used to ease communication between different computational infrastructures. A recent example is the FHIR-based German Medical Informatics Initiative Core Dataset (MII-CDS), which is specifically designed for German university hospital patient data, to enhance its usability in joint research endeavors [19]. In addition, Common Data Elements (CDEs) for RD registration have been introduced [20] e.g., via the European Platform for Rare Disease Registration (ERDRI) [21], the French National Plan for Rare Diseases, [3, 22], the effort for a minimum dataset for rare diseases [23], and more recently domain-specific CDEs for RDs registries [24]. Exemplarily, Mullin et al. [25] describe the development and application of standardized data structures for rare diseases, specifically focusing on Duchenne muscular dystrophy (DMD) and Huntington's disease (HD), using Clinical Data Interchange Standards Consortium (CDISC) therapeutic area user guides. These guides support the mapping process for diverse clinical data into standardized formats, ensuring consistency and enabling comprehensive data analysis across studies. The model emphasizes the creation of formalized structures for clinical data, which include predefined measurements and controlled terminology. This model aids in streamlining the regulatory approval process by providing standardized data that can be more easily reviewed and analysed by authorities like the Food and Drugs Administration (FDA).

Kaliyaperumal et al. [20] discuss the development of semantically grounded models for RD data, aimed at enhancing interoperability among disparate RD registries in Europe. The project is part of the European Joint Programme on Rare Diseases (EJP RD), which aligns with the European Platform on Rare Disease Registration (EU RD Platform). The researchers developed semantic models for Common Data Elements (CDEs) using the Semantic Science Integrated Ontology (SIO) as the core framework. These models map the data elements and their possible values into established domain ontologies, such as the Orphanet Rare Disease Ontology and Human Phenotype Ontology.

The work of Kim et al. [26] focuses on a comprehensive model for improving semantic interoperability in healthcare by extending the capabilities of CDEs. The model outlines significant enhancements, including the introduction of new semantic types and constraints for CDEs to address limitations in existing standards for managing complex clinical data. They introduce hybrid atomic, repeated, and dictionary composite CDEs to support complex data structures and relationships within clinical documentation. The authors have tried to implement constraints, such as 'ordered', 'operated', 'required', and 'dependent' to enhance data integrity and semantic evaluation. Assessment of the model is done using clinical documents from five teaching Korean hospitals and data from FHIR resources and the Medical Information Mart for Intensive Care (MIMIC-III) database, demonstrating improved data reuse and semantic interoperability.

In the RD field however, data standardization still poses a major limitation [14], data harmonization concepts like common data models (CDM), are being more widely employed to address the issue of data harmonization with an increased emphasis on genomic and phenotypic data. In general, a CDM can harmonize data from disparate sources, by utilizing communication (e.g., FHIR) and semantic (e.g., ICD-10, SNOMED) standards, enabling operations and analyses solely based on standard methods [27]. One very promising approach in addition to the aforementioned ones is the Observational Medical Outcomes Partnership (OMOP) CDM from the Observational Health Data Sciences and Informatics (OHDSI) community, which comes with FAIR compliance, an international community, and ready-to-use tools for data integration and analysis [28–30]. Compared to other CDMs like Informatics for Integrating Biology & the Bedside (i2b2), OMOP CDM offers broader terminology coverage, enabling data harmonization from different sources with minimal loss of data [28, 31, 32].

The adaptation of OMOP terminology for RD has already been shown to be essential for improved patient care [33]. Importantly, OMOP CDM also supports genomic data representations [28, 34, 35], and works are underway to integrate Human Phenotype Ontologies (HPO) [36] and Orpha Codes [37], further enriching the terminology coverage of OMOP CDM and thus creating an increased interest in the domain of RD [33, 38, 39]. In summary, all necessary tools and approaches are on the way for dedicated use and, if conducted correctly, a large-scale investigation of RD can be designed and conducted via OMOP CDM.

Our study is taking up these developments and demonstrates how customized CDMs and underlying data structures can be designed to facilitate and accelerate the understanding of RDs. In particular, we provide insights into current computational methods used for modulating standardized concepts, such as laboratory findings and medication, but also more specific data, such as genotypes to investigate different clinical phenotypes. We conducted this study as a practical continuation and application of the derived results made by our recently published scoping review on development methods of CDMs in Healthcare [40] in a larger project entitled SATURN (“Smart physician portal for patients with unclear disease”) [41] dealing with RDs in the fields of endocrinology, gastroenterology, and pneumonology. In addition, our evaluation use case domain of hematology (acute myeloid leukemia) data was transformed to the CDM for external validation of the model.

### Contribution of this study

Our primary objective is to outline a customization process aimed at identifying and systematically modeling pertinent data from patients with RD. The outcome customized CDMs, will be serving as a foundational source for transferring observational data into the OMOP CDM. This intermediate step is instrumental in establishing a shared knowledge base between medical experts and data scientists, ensuring simultaneous enhancement of semantic and syntactic interoperability.

## Methods

### Clinical use cases and their underlying data

Based on our available clinical resources and datasets, we have exemplarily chosen four RD domains, to begin with, namely endocrinology, gastroenterology, pneumonology for general CDM customization and development, and hematology as a proof-of-concept domain in particular for genotype information. The study characteristics including the inclusion criteria, and International Statistical Classification of Diseases and Related Health Problems, 10th revision, German Modification (ICD-10-GM) codes are shown in Table 1, respectively. As a study of this nature relies on the availability of data, which is typically scarce when it concerns RDs, our selected list of diseases encompasses a mixture of both rare and more common conditions. Additionally, the SATURN project does not only focus on RDs, but also unclear diseases. We have therefore decided to focus on a list of phenotypes that can be caused by rare and non-rare diseases. In endocrinology, this resulted in the symptom complex hyper- and hypothyroidism with various diseases, some of which are rare (e.g. TSH-oma, thyroid receptor resistance), while others are common (autoimmune thyroiditis).

**Table 1:** Study characteristics including study population, inclusion criteria, and number of patients in each one of our cohorts

Study population	Inclusion criteria	Number of patients period 2015–2022 from two German university hospitals
Full or partial inpatient cases of adult patients	Patients are older than 18 years at the time of (visit) admission	–
Cases of patients in the period from 2015–2022	The admission and discharge dates of the patients are in the period from 01/01/2015 to 12/31/2022	–
Cases of patients of the selected endocrinological diseases	Patients diagnosed with one of the following conditions: Clinical hyperthyroidism (E05.-), incl.: Latent hyperthyroidism Manifest hyperthyroidism Central hyperthyroidism Graves’ disease Iatrogenic (therapy-induced) hyperthyroidism Autonomy of the thyroid gland Amiodarone-induced hyperthyroidism Pituitary thyroid receptor resistance TSH-oma Thyroiditis de Quervain (E06.1) Hypothyroidism incl.: Aplasia of the thyroid gland (E03.1) Iatrogenic (therapy-related) drug-induced (E03.2) Iatrogenic hypothyroidism/condition after surgery (E89.0) Autoimmune thyroiditis (E06.3) Secondary & tertiary hypothyroidism (E23.0) Sarcoidosis (D86.-)	41,559
Cases of patients of the selected gastroenterological diseases	Patients diagnosed with one of the following conditions: Acute viral hepatitis A (B15.-) Acute viral hepatitis B (B16.-) Other acute viral hepatitis (B17.-) Chronic viral hepatitis (B18.-) Wilson’s disease (E83.0) Hemochromatosis (E83.1) Alpha-1 antitrypsin deficiency in adults (E88.0)	1324
Cases of patients of the selected pneumonological diseases	Patients diagnosed with one of the following conditions: Bronchial carcinoma (C34.-) Tuberculosis (A15.-) Sarcoidosis (D86.-) Influenza (J10.-)	17,141
Cases of patients of the selected hematological diseases	Patients diagnosed with acute myeloid leukemia (C92.0)	1673
Sum		61,697

### Customized RD-CDM

Given the wide range of existing information models, legislative regulations for data exchange and terminology standards in the medical domain, it is crucial to know and leverage their features and combine them effectively, especially when dealing with RD. Therefore, we utilized semantic standards, such as ICD-10-GM, which is the German modified version of the World Health Organization’s ICD-10, to define the inclusion criteria in the study.

Moreover, to ensure the reproducibility of the anticipated CDM workflow of a novel study, it is essential to choose a widely adopted and commonly used data standard, which ensures semantic and syntactic interoperability. Therefore, we have utilized the FHIR format in its German University Hospital-centric MII-CDS as a central point for our data transfer [19]. The MII-CDS basic modules include “Person”, “Case”, “Diagnosis”, “Procedure”, “Laboratory findings”, and “Medications” [19]. The MII-CDS profiles are comparable to FHIR because they are built using FHIR profiles.

Finally, to facilitate an international usage of the data in large scale, multi-center studies, we have utilized OMOP, which in addition to standardized semantics and syntax on an international level, also motivates the usage of standard vocabularies (e.g., SNOMED CT).

#### CDM development

**Fig. 1:**
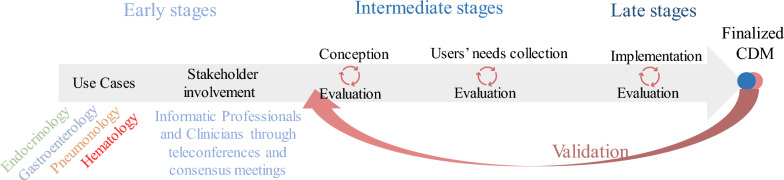
Steps taken in the development of our CDM for rare diseases. The three phases (early: idea, use case definition, and stakeholder identification, intermediate: conception and users’ need collection, and late: implementation, stages) include one or multiple iterative steps. The four use cases were defined first based on available resources and stakeholders were then identified accordingly. We decided on the means of stakeholder involvement in the process as well. The conceptualization step was part of the intermediate phase in an evaluative process before the necessary information was then gathered from medical experts, clinical guidelines, and literature. We then finalized our CDM by mapping the elements to standardized concepts and formalizing them into separate modules

Our recently published scoping review [40], describes an analysis of 1309 articles spanning the last 20 years (2000–2022) on CDM development for the health domain and an extensive metadata extraction of 59 articles. The extraction step was specifically focused on stakeholder involvement, the methods employed for their engagement, and the detailed description of the development process of the respective CDMs. As a result, we have delineated the process of customizing a CDM for RD into three distinct phases: conception, collection of users' needs, and implementation, as illustrated in Fig. 1. We followed these three phases in our current study to obtain a list of elements for our focused RD domains and structured all elements into a customizable CDM.

#### Customization need

Building on the insights from our scoping review [40], we recognized the importance of involving stakeholders early on in our study. Thus, we conducted consensus meetings and teleconferences with an interdisciplinary team of researchers, medical experts, and data scientists to develop and evaluate the customized CDM. Throughout the process, we ensured that at least one clinician for each domain was involved in the upcoming stages i)–iv).

1. **Conception:** To accommodate the unique goals of individual projects, creating a project-specific CDM is crucial for supporting diverse clinical investigations. Importantly, the development of such a customized CDM opens the possibility for its utilization across multiple projects, emphasizing efficiency and fostering a shared knowledge base through collaborative efforts of medical experts and data scientists in identifying and systematically modeling relevant data. Thus, a list of diseases with similar phenotypes and clinical procedures, which could cause confusion while diagnosing, was compiled by medical experts. We aimed to develop a customized CDM to serve as source data for the OMOP CDM. Ensuring accurate transfer, the adapted CDMs closely align with the data and information model of the OMOP CDM.
2. **Users’ needs collection:** Based on medical literature, clinical guidelines, and medical domain expertise, we created a list of required clinical phenotypic and genotypic elements, laboratory findings, medications, and procedures for diagnosis and therapy of each disease. The expert knowledge was formalized by the medical data scientist in the form of pre-defined list of elements modules using an entity-relationship model. An entity-relationship model defines an interrelated set of elements and the relationship that can exist among them [42].
3. **Implementation:** This stage involves formalizing the data model, aligning it with OMOP, and integrating data through ETL. The predefined modules from individual use cases are transformed into MII-CDS modules. We assessed the feasibility of mapping formalized information into both FHIR and OMOP CDM. The MII-CDS serves as the foundation for the FHIR-to-OMOP [43] ETL process, eliminating the need for manual mapping, as the existing ETL process seamlessly transfers data from FHIR to OMOP, ensuring semantic and syntactic mapping. However, manual mapping was required for genotype and phenotype elements, as they are not covered in the FHIR-to-OMOP ETL. An additional ETL process (Genotype/Phenotype-to-OMOP) was developed for their manual mapping to unique concept IDs in OMOP. This ETL was tested using our proof-of-concept domain hematology data to our OMOP based RD-CDM.
4. **Evaluation:** The modules were then assessed by the medical experts in an iterative process equally involving medical experts and data scientists. The experts’ comments and input were integrated into the modules by the data scientist until a final consensus model was reached and agreed upon by all stakeholders. Once the modules were finalized, a second independent medical expert reviewed and evaluated the completeness and correctness of the included elements.

### CDM data integration for development domains and the validation domain

**Fig. 2:**
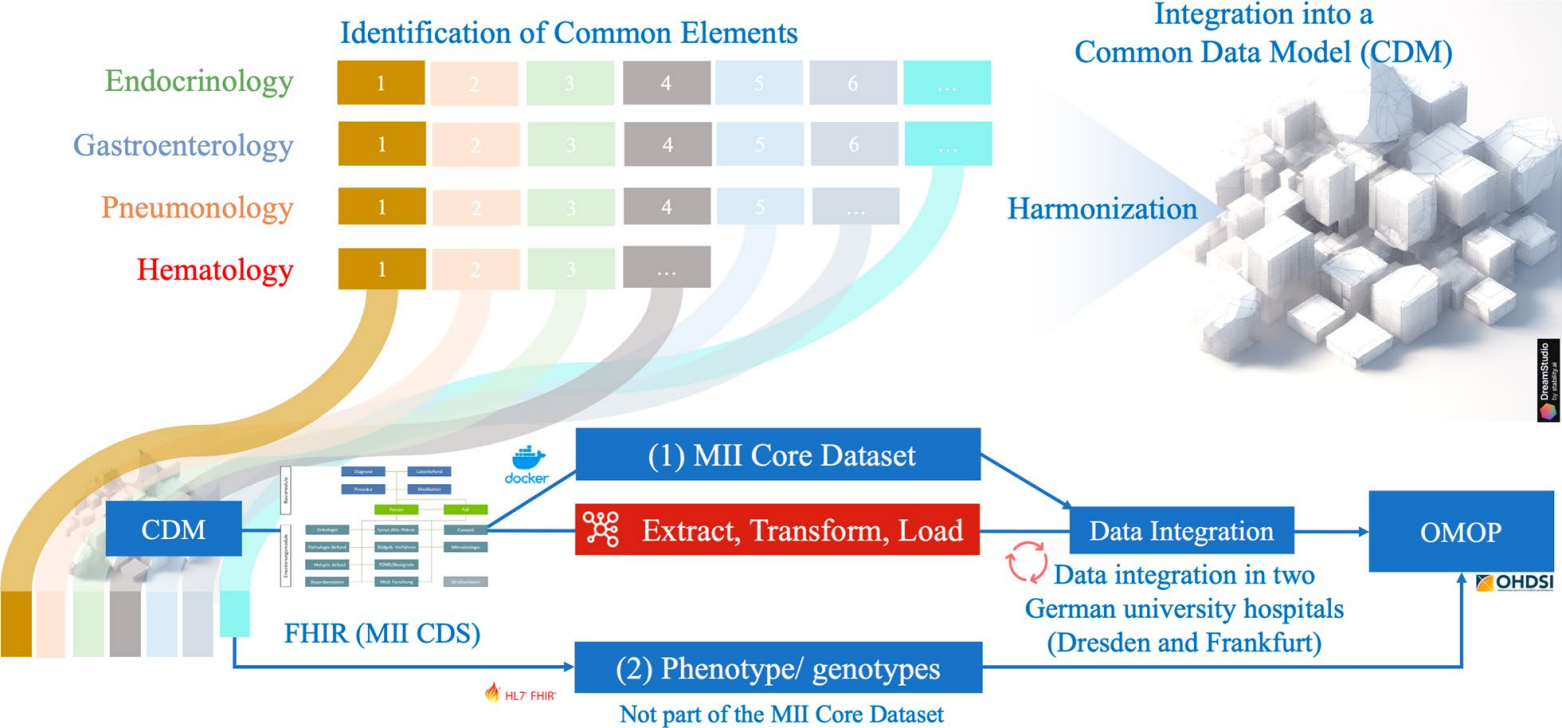
Data integration steps from the customizable RD-CDM to OMOP CDM: the developed RD-CDM was mapped to the FHIR communications standard (MII-CDS). In the next step, using the two ETL routes the integrated modules were then transformed to OMOP. The “Person”, “Case”, “Diagnosis”, “Procedure”, “Laboratory findings”, and “Medications” are part of the MII-CDS, which was also transformed using the ETL process from FHIR to OMOP. The Phenotype and Genotype information are transformed using a direct ETL process from RD-CDM to OMOP CDM

To achieve syntactic and semantic interoperability, we opted to transition our developed CDM to OMOP to facilitate streamlined data analysis using existing Machine Learning tools (e.g., PLP, HADES) [44, 45] to enable larger multi-center studies. Our utilized FHIR-based MII CDS basic modules have already been mapped to OMOP CDM in recently published works (FHIR-to-OMOP) [43]. However, the phenotypic and genotypic entities are not part of the MII CDS basic modules. Therefore, we directly mapped those elements to OMOP CDM concept ids. Thus, we developed the Genotype/Phenotype-to-OMOP ETL [46] processes using Pentaho Data Integration [47] to transfer elements not included in MII CDS basic modules to OMOP CDM. MII CDS basic modules to OMOP ETL should be executed before the Genotype/Phenotype-to-OMOP ETL processes to guarantee referential integrity between the patient ID and visit IDs. As HPO is not currently offered as an OMOP-compliant vocabulary, the required HPO terms are loaded into the SOURCE_TO_CONCEPT_MAP as individual source codes by the ETL process. The three ETL processes are used together to transfer the information for endocrinology, gastroenterology and pneumonology to the customized CDM from two university hospitals (Dresden and Frankfurt) and hematology dataset from the university hospital Dresden to OMOP CDM. The process is also highlighted in Fig. 2.

### Derived blue-print and lessons learned

Drawing insights from the development and implementation phases, and collaboration with various stakeholders and interdisciplinary experts involved, we have compiled a list of crucial steps that will facilitate the customization of our CDM beyond the presented RD. Additionally, we have included key lessons learned that emerged throughout the entire process.

## Results

### Use case-specific data models

Our study encompasses nine disease groups from the endocrinology domain, two from gastroenterology, four from pneumonology, and one from the hematology domain. Table 2 presents the list of diseases along with their respective ICD-10-GM and Orpha codes [48]. In total 61,697 patients were included in this study.

**Table 2:** List of diseases in three development domains (endocrinology, pneumonology, and gastroenterology) of rare diseases and the validation domain (hematology)

Domain	Disease Groups	Diseases	ICD-10-GM	ORPHAcode
Endocrinology	Hyperthyroidism	Clinical hyperthyroidism	E05.9	ORPHA:181,399
		Latent hyperthyroidism	E05.9	
		Manifest hyperthyroidism	E05.9	
		Graves' disease	E05.0	ORPHA:525,731
		Iatrogenic (therapy induced)	E05.8	ORPHA:576,379
		Unifocal iatrogenic (therapy-induced)	E05.8	
		Autonomy of the thyroid gland: multifocal autonomy of the thyroid gland	E05.8	ORPHA:3143
		Autonomy of the thyroid gland: disseminated autonomy of the thyroid gland	E05.8	–
		Thyroiditis de Quervain	E06.1	–
		Amiodarone-induced hyperthyroidism type I	E05.8	–
		Amiodarone-induced hyperthyroidism type II	E05.8	–
		Pituitary thyroid receptor resistance	E05.8	–
		TSH-oma	D35.2	ORPHA:424
	Hypothyroidism	Clinical hypothyroidism	E03.-	–
		Aplasia of the thyroid gland	E03.1	–
		Iatrogenic (therapy-related) drug-induced	E03.2	–
		Iatrogenic hypothyroidism/condition after surgery	E89.0	–
		Autoimmune thyroiditis	E06.3	–
		Secondary & tertiary hypothyroidism	E23.0	–
		Sarcoidosis	D86.-	–
Gastroenterology	Viral Hepatitis	Hepatitis A	B15.0	–
		Acute hepatitis B	B16.0	–
		Acute hepatitis C	B17.1	–
		Acute hepatitis E	B17.2	–
	Hereditary liver diseases	Hemochromatosis	E83.1	–
		Wilson's disease	E83.0	–
		Alpha-1 antitrypsin deficiency in adults	E88.0	–
Pneumonology	Solid malignancy	Solid malignancy (Bronchial carcinoma)	C34.-	–
	Tuberculosis	Tuberculosis	A15.-	–
	Sarcoidosis	Sarcoidosis	D86.-	–
	Viral infections	Influenza	J10.0	–
Hematology	Cancer	Acute myeloid leukemia (AML)	C92.0	ORPHA:319,465

### Entity relationship model to aggregate a list of relevant elements

**Fig. 3:**
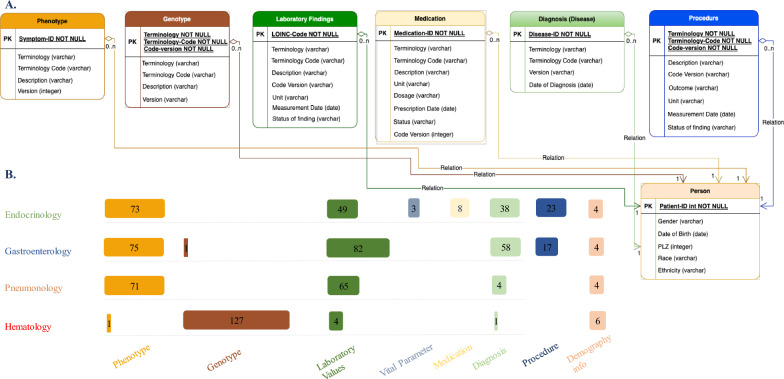
**A** Developed CDM modular structure. It includes seven tables to store all the clinical, demographic, and genetic information. The (0…n) shows the relationship between the tables. E.g., for each unique patient id in the person table there can be zero or multiple (0 to n) entries in the other tables. **B** Shows the included use case specific sets of elements included in the CDM. The numbers on the bars are equal to the number of elements in each of these sets. e.g., we have included four demographic info (gender, date of birth, age at diagnosis, and postal code) for endocrinology, gastroenterology, and pneumonology. For hematology, we added race and ethnicity information additionally as it was available as part of the provided data. (PK = primary key)

Figure 3A illustrates an entity-relationship model that shows a consolidated list of elements from the diseases used to develop the customizable RD-CDM structure. Figure 3B illustrates a list of attributes and their formalization on the CDM modules. Each bar in the figure is labeled with a number indicating the quantity of elements for each category in our domain specific data models. The complete list of these elements can be viewed in our CDM model as Additional file 1 and on our GitHub page [46].

### Joint symptoms within domains

Table 3 illustrates the overlapping symptoms and their corresponding HPO codes across the diseases within each domain. Our hematology domain, which features Acute Myeloid Leukemia (AML) and includes fever as the primary symptom, is not presented in this table due to its limited scope.

**Table 3:** Overlapping symptoms within the domain. In the Diseases column, all diseases that include the corresponding symptoms are listed

Group	Symptoms	HPO Code	Diseases
Gastroenterology	Icterus or Jaundice	HP:0000952	Viral hepatitis (A, B, C, D, E), alpha-1 antitrypsin deficiency, De Quervain's thyroiditis, amiodarone-induced hyperthyroiditis
	Skin rash	HP:0000988	Viral hepatitis (A, B, D and E)
	Ascites	HP:0001541	Viral hepatitis B, C, hemochromatosis, Wilson’s disease, and alpha-1 antitrypsin
	Gastrointestinal bleeding	HP:0002239	
	Fever	HP:0001945	Viral hepatitis, A, B, C, D and E
	Vomiting	HP:0002013	Viral hepatitis A, C, and E
	Diarrhea	HP:0002014	
	Nausea	HP:0002018	
	Hepatic encephalopathy	HP:0002480	Viral hepatitis B, C, hemochromatosis, Wilson’s disease, and alpha-1 antitrypsin deficiency in adults
	Arthralgia	HP:0002829	Viral hepatitis B, C, D, and hemochromatosis
	Myalgia	HP:0003326	Viral hepatitis B, C, D,
	Poor appetite	HP:0004396	Viral hepatitis B, D
	Fatigue	HP:0012378	Viral hepatitis B, D, Wilson’s disease, and alpha-1 antitrypsin deficiency
	General (health) Condition Reduction	HP:0033666	Viral hepatitis (A, B, C, D and E)
	Upper abdominal pain	HP:0410019	
Pneumonology	Dyspnea	HP:0002094	Solid malignancy, sarcoidosis general, sarcoidosis acute form
	Chest pain	HP:0100749	
	Weight loss	HP:0001824	Solid malignancy, tuberculosis, and sarcoidosis both form acute and chronic
	Fever	HP:0001945	Solid malignancy, tuberculosis, sarcoidosis both forms acute and chronic, influenza
	Fatigue	HP:0012378	
	Cough	HP:0012735	Solid malignancy, tuberculosis, sarcoidosis general, sarcoidosis acute form, viral infection e.g., influenza, sarcoidosis chronic form
	Leukocytosis	HP:0001974	Tuberculosis, sarcoidosis general, viral infection e.g. influenza
	Headache	HP:0002315	Solid malignancy, influenza
	Poor appetite	HP:0004396	Tuberculosis, solid malignancy, sarcoidosis acute form
	Abnormal blood sodium concentration	HP:0010931	Solid malignancy
	Night sweats	HP:0030166	Solid malignancy, tuberculosis
Endocrinology: Hyperthyroidism	Palpitations	HP:0001962	Clinical hyperthyroidism, latent hyperthyroidism, manifest hyperthyroidism, Graves' disease, iatrogenic (therapy-related), unifocal autonomy of the thyroid gland, multifocal autonomy, disseminated autonomy, De Quervain's thyroiditis, amiodarone-induced hypothyroidism type 1 and 2, Pituitary thyroid receptor resistance, TSH-oma
	Anxiety	HP:0000739	
	Hyperhidrosis	HP:0000975	
	Hand tremor	HP:0002378	
	Restlessness	HP:0000711	
	Irritability	HP:0000737	
	Diarrhea	HP:0002014	
	Weight loss	HP:0001824	
	Abnormal eating behavior	HP:0100738	
	Heat intolerance	HP:0002046	
	Sleep disturbance	HP:0002360	
Endocrinology: Hypothyroidism	Abnormality of the menstrual cycle	HP:0000140	Hypothyroidism in general, secondary hypothyroidism: originating from the anterior pituitary lobe, tertiary hypothyroidism: originating from the hypothalamus
	Depression	HP:0000716	
	Gynecomastia	HP:0000771	
	Dry skin	HP:0000958	
	Hyporeflexia	HP:0001265	
	Bradycardia	HP:0001662	
	Pericardial effusion	HP:0001698	
	Constipation	HP:0002019	
	Hypotension	HP:0002615	
	Increased body weight	HP:0004324	
	Hyperlipoproteinemia	HP:0010980	
	Fatigue	HP:0012378	
	Diminished ability to concentrate	HP:0031987	
	Coldness	HP:0033850	
	Pretibial myxedema	HP:0200028	Hypothyroidism in general, secondary hypothyroidism: originating from the anterior pituitary lobe

**Fig. 4:**
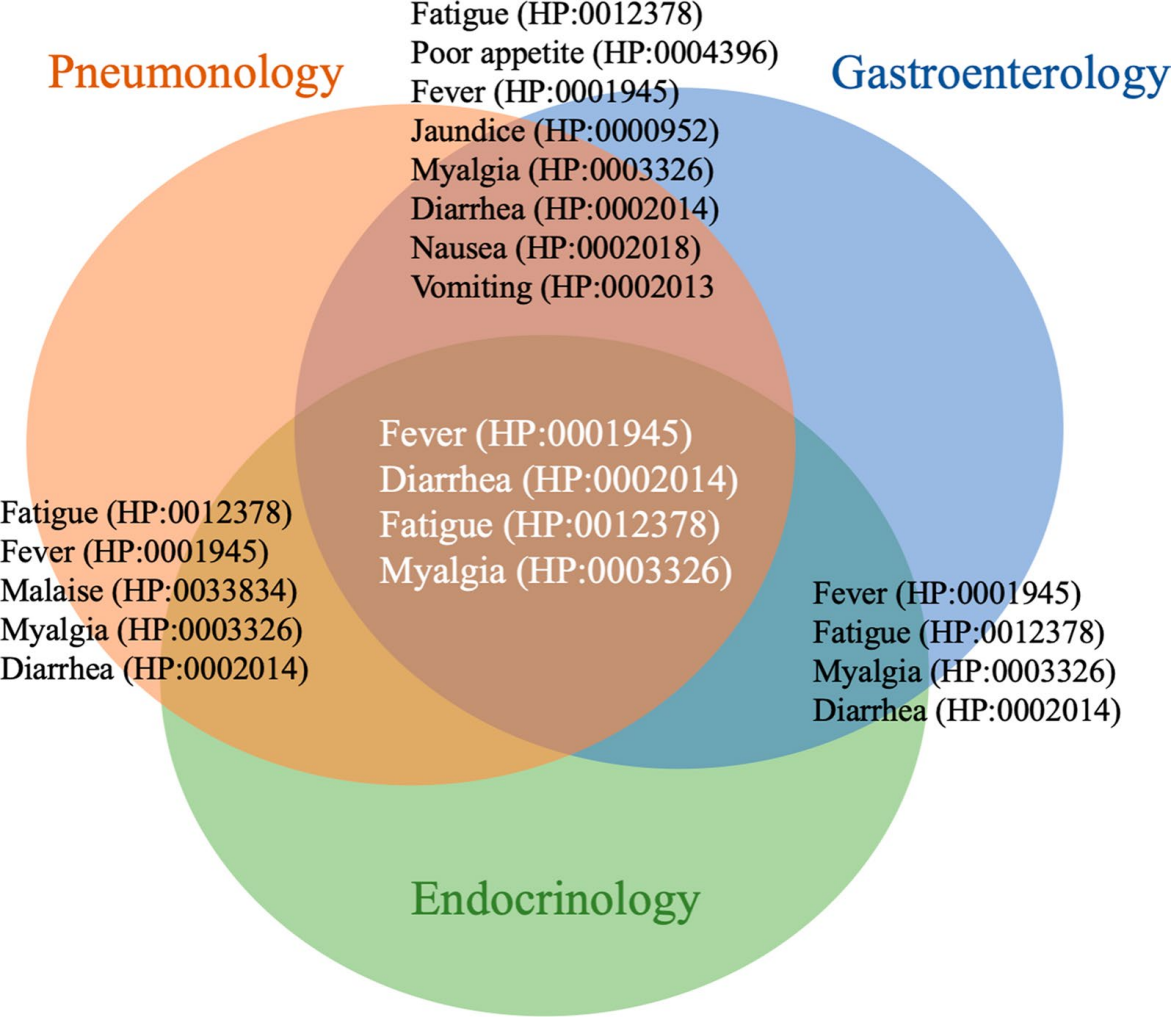
Joint symptoms between groups. Shared between all groups are: fever, diarrhea, fatigue, and myalgia. Between pneumonology and gastroenterology fatigue, poor appetite, fever, jaundice, myalgia, diarrhea, nausea, and vomiting are shared. The pneumonology and endocrinology symptoms that overlap are fatigue, fever, malaise, myalgia, and diarrhea. The overlapping symptoms between gastroenterology and endocrinology however are, fever, fatigue, myalgia, and diarrhea

Fever is the only symptom that appears across all four domains we studied, including hematology. Furthermore, symptoms such as diarrhea, fatigue, and myalgia are shared among the endocrinology, gastroenterology, and pneumonology domains. Table 3 also highlights symptoms that overlap between just two domains, as depicted in Fig. 4.

### Mappings and ETL processes to streamline RD-CDM applicability

Given our use of the FHIR MII-CDS as an intermediary step, we mapped our data entities to corresponding modules within MII-CDS. Except for phenotypes and genotypes, we successfully aligned all other entities to MII-CDS modules. Due to the availability of existing ETL and FHIR-to-OMOP mapping processes, there was no need for manual mapping for these entities.

For the mapping of phenotypic and genotypic data, we manually aligned our model’s entities to OMOP concept IDs utilizing the terminology search engine, Athena [49]. As detailed in Table 4 the demographic information can be aligned to OMOP using Gender Concepts. The diagnosis can be mapped using ICD-10-GM and SNOMED concepts. For procedures, Operation and procedure codes (OPS) [50] and SNOMED concepts are available. The laboratory findings are mapped using Logical Observation Identifiers Names and Codes (LOINC) [51] concepts and the unit codes are documented using Unified Code for Units of Measure (UCUM). Medication can be integrated using RxNorm [52] and Anatomical Therapeutic Chemical (ATC) [53] concepts. Additionally, most of the genotype elements can be mapped using OMOP Genomic terminology released from OHDSI [34].

**Table 4:** Mappings between the RD-CDM, FHIR modules, and OMOP tables as well as the terminology used for semantic mapping

RD-CDM	FHIR (MII-CDS)	OMOP tables	Vocabulary
Person	Person	Person	SNOMED; Gender
Diagnosis	Diagnosis	Condition_occurrence, procedure_occurrence, measurement, or observation	SNOMED; ICD-10-GM; OrphaCode
Procedure	Procedure	Drug_exposure, procedure_occurrence, measurement or observation	SNOMED; OPS
Laboratory Findings	Laboratory test results	Observation, measurement or procedure_occurrence	LOINC; UCUM
Medication	Medication	Drug_exposure or observation	RxNorm; ATC
Genotype	–	Observation; measurement	OMOP Genomic
Phenotypes	–	Observation; measurement	HPO; SNOMED; LOINC

### Hematology as a validation use case

The *Genotype-to-OMOP* ETL process designed to handle a hematology dataset comprising 1,674 patients and 124 clinical and gene mutation data features, was rigorously tested and is available for download and modification from GitHub [46]. We predominantly used OMOP Genomics terminology to map our gene mutation elements to OMOP concept ids. Additionally, SNOMED, LOINC, the Diagnosis-related group (DRG) [54], and UCUM were employed for this dataset. We were able to map and integrate our data entities with 100% success from a CSV file to our previously developed OMOP instance. However, in certain instances, it was necessary to map multiple gene mutation details to a single OMOP concept id. An example is the “35,948,202—CEBPA (CCAAT enhancer binding protein alpha) gene variant measurement, that we used to transform, “CEBPA.bZIP”, “CEBPA.bZIP.inframe”, “CEBPA.TAD”, and “CEBPASTAT” information of the patients to OMOP. The complete mapping table is also available on GitHub [46].

### Comparison of RD-CDM against the minimum dataset for Rare Diseases

**Fig. 5:**
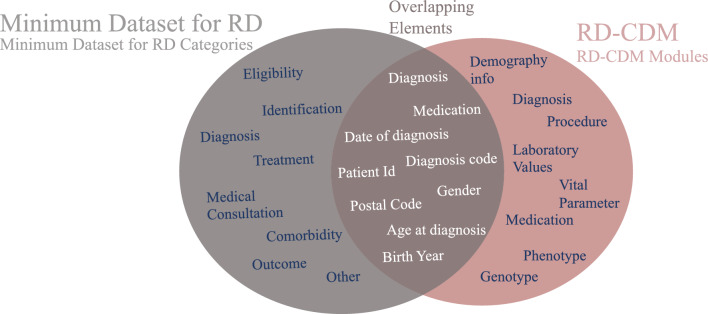
This figure shows a comparison of elements in the minimum dataset for Rare Diseases (RD) and the OMOP based Rare Diseases common data model (RD-CDM). The left circle shows the categories of the Minimum Data for RD and the right circle shows the RD-CDM modules before being mapped to OMOP CDM. The overlapping elements from different modules of RD-CDM to the minimum Dataset for RD Categories are shown in the middle

In comparison to our model, the minimum dataset for rare diseases [23] includes ten groups of elements. They include Eligibility, Identification, Diagnosis, Treatment, Medical Consultation, Comorbidity, Outcome, and others. Some of the elements of the Identification group in the minimum dataset, such as Ethnic background, is not part of the data collected in German hospitals. Some others, such as the patient id or the address of residence could only be included in our data model in a pseudonymized format considering patient privacy. Furthermore, the minimum dataset for RD included eligibility and identification elements that are important to identify patients on the registry level. On the contrary, the RD-CDM is the basis of a diagnostic support system developed in the SATURN project [41], which is why most of our included elements are actually focused on diagnostics. The overlapping elements between the RD-CDM and the minimum dataset for RD are shown in Fig. 5.

### Customize your RD-CDM-based study in six brief steps

Here, we provide a brief description to demonstrate how the RD-CDM can be reused on other data and medical domains for RD.

1. Definition of the use case(s).
  Definition of modules that are needed or are of interest to investigate the research hypothesis at hand.
  Identification of all relevant elements within the modules that contribute to answering the research question.
2. Definition of the stakeholders’ circle based on the use case(s).
  After the definition of the required modules and elements, they should be communicated and iterated with other medical experts to check for their availability at each and every participating study site.
  It is essential to take into account the perspective of the data providers. In the described study, it becomes crucial for each participating site to reach out to the respective data integration center. This step is necessary to verify the feasibility of automatically retrieving the requested data, potentially in standard formats such as FHIR or with associated terminologies like SNOMED.
  Given the sensitive nature of medical data, it is imperative to adhere to ethical standards, prioritize data security, and safeguard privacy. In light of these considerations, the responsible organizational entity must initiate contact with legal authorities to ensure a comprehensive and legally sound approach to managing and addressing the data's sensitivity.
3. A list of diagnostic entities may be created together with the stakeholders for the targeted use case(s).
  Once an iteration between all stakeholders and the initial set of modules and elements has been done, one may continue with the full or restricted list of data elements for the study.
4. The use of use case-specific entities may be mapped to the modules of RD-CDM.
  Individual data elements from the specific data source should be directed towards the unified CDM structure (common target data structure, jointly used by all involved study sites).
5. ETL processes, such as our FHIR-to-OMOP ETL process, should be used for transferring data to OMOP in the CDM dedicated “*Person*”, “*Diagnosis*”, “*Laboratory findings*”, “*Procedure*”, and “*Medications*” modules to unify the data elements among all participating study sites.
6. The “Genotype” and “Phenotype” modules could use direct ETL processes from CSV to OMOP CDM to transfer the data into OMOP.
  An example of such an individual ETL process can be obtained from our GitHub instance (Genotype/Phenotype-to-OMOP*) *[46]*.*

## Discussion

Despite efforts to standardize infrastructure and integrate large-scale data in clinical research, challenges remain in personalized medicine [55, 56], particularly for the documentation of genotypes, phenotypes, and clinical data for RD [14, 55, 57]. Comprehensive documentation is crucial for patient recruitment, standard care monitoring, natural history assessment, genotype–phenotype correlation analyses, and disease burden evaluation, ultimately enhancing our understanding of RD [33]. Nevertheless, the planning, design, maintenance, and sustainability of such large-scale medical studies need to be improved and streamlined [14].

In this study, we illuminated the impactful role of customized CDMs, particularly in the context of forming a collaborative foundation between medical experts and data scientists within domain-specific projects. By modeling and utilizing these tailored CDMs, we achieved two critical objectives: (a) establishing a shared knowledge base that enhances communication and understanding between medical experts and data scientists, and (b) streamlining the harmonization of source data. This harmonization, in turn, facilitates a seamless transfer to the internationally recognized research database, OMOP CDM, underscoring the versatility and effectiveness of our approach in advancing multi-center data-driven studies.

In particular, we developed a customized RD-CDM based on the OMOP information model and utilizing the FHIR communication standard. The combination of both enabled us to efficiently utilize the already existing ETL processes [43] for the mappings to OMOP. The final CDM consists of several modules, including “*Person*”, “*Diagnosis*”, “*Procedure*”, “*Laboratory findings*”, and “*Medications*”. These modules are also part of the FHIR MII CDS. Moreover, two additional modules, “*Genotype*” and “*Phenotypes*”, were added to the customizable CDM structure to better capture the unique characteristics of RD.

### Use case-specific application examples

Some diseases from disease groups, such as hyperthyroidism and its distinct etiologies can present with very close similarities and thus be challenging to differentiate solely based on clinical data. Our customizable RD-CDM can improve this, which will be further evaluated during the SATURN project, which seeks to assist the diagnostic process. Additionally, the RD-CDM-based data will be used in analytical studies to answer clinical questions. Here, the inclusion of the Genotype and Phenotypes modules into the CDM structure allows for the capture of more detailed and specific information related to RD that may not be available in other standard CDMs.

The CDM has additional advantages applicable to all RD groups. These encompass the formulation of specific clinical questions for international and multi-center studies, including the prospective integration of genotype data, particularly concerning the potential incorporation of Human Genome Variation Society (HGVS) nomenclature [58] with a focus on exploring genotype–phenotype correlations.

Moreover, the CDM incorporates information about certain procedures by using OPS. This enhances research by facilitating the establishment of connections between imaging results with genotype information. Use case-specific applications would include the matching of genotype with liver imaging data in hemochromatosis or with sonography and scintigraphy data in diseases that affect the thyroid gland.

Furthermore, the CDM could be helpful for the clustering of subgroups for certain RD based on phenotype or genotype. Within the field of endocrinology, it allows for differentiation based on laboratory parameters, enabling the distinction between latent and manifest forms of hypothyroidism or autoimmune forms, depending on the presence of specific antibodies. Tuberculosis can affect various organs, leading to different phenotypic expressions. Using a suitable CDM, patients with diverse manifestations could be categorized based on their phenotypic profiles. In the context of hemochromatosis, it allows subgroup formation based on genetic factors. When genetic information is unknown or inconspicuous, the CDM can facilitate the creation of subgroups based on symptoms only.

These benefits also encompass the collection of patients with similar Variants of Uncertain Significance (VUS) and the comparative analysis of symptoms and phenotypes, facilitating their categorization into subgroups for research purposes or potential reclassification. Additionally, the potential exists for investigating the co-occurrence of specific RD and assessing whether certain mutations may render individuals more vulnerable to other conditions, such as heightened infection risk for hepatitis.

In cases of incomplete penetrance of certain mutations, e.g. in hemochromatosis, the CDM is valuable for aggregating asymptomatic or minimally symptomatic patients based on their genotype for long-term risk assessment. It also supports the categorization of asymptomatic family members who have been subsequently examined as a distinct group.

Moreover, the flexibility of tailoring therapy studies according to the specific genotype for gene therapy is a valuable prospect. A coherent mapping of clinical information and underlying disease biology as genotype–phenotype maps may not only aid in identifying disease categories with different clinical presentations but also tailor personalized treatment approaches to patient biology. Especially in high-stakes environments, such as AML treatment, where fast and accurate diagnosis, as well as rapid treatment initiation according to molecular subtypes, is crucial [59], a better understanding of genotype–phenotype associations in multi-center data-driven studies holds the promise of improved treatment outcomes with targeted therapies, while avoiding resistance and relapse.

The benefits of RD-CDM are therefore evident for all use cases. Overall, the CDM significantly enhances our ability to comprehensively study and understand the complexities of RDs regardless of the focus domain.

### General benefits of using CDMs

Integration of heterogeneous data is a ubiquitous topic in modern medicine. This arising large variety of data has the potential to be used for deriving insights about the different aspects of care and lead to improvements in health care. Yet, challenges, such as identifying and accessing relevant data, the association between different data sources, and ensuring the data quality given the structural variations amongst data sources are posing a barrier [60, 61]. That is why data is still sparse, especially more patient-specific data, such as genotypes and phenotypes, which are especially important for RDs. Therefore, the development of a comprehensive CDM tailored to the unique domains of RD is of importance. Our RD-CDM, built on the foundation of OMOP, serves as a framework for standardizing additional data components across multiple domains. It is suitable for usage in analytic processes involving machine learning and statistical models. In addition, because OMOP is well established as a research data model, our CDM facilitates collaboration with different research groups at different sites on an international level, effectively addressing the challenge of data scarcity, which is particularly critical in the field of rare diseases.

While Composite CDEs and OMOP CDM both aim to standardize healthcare data, they serve different purposes and contexts. Composite CDEs are more focused on detailed and standardized data collection within specific studies or trials, ensuring consistency and comprehensiveness for particular clinical concepts. In contrast, OMOP CDM offers a flexible, scalable framework for integrating and analyzing diverse healthcare data across multiple settings, supporting large-scale observational research and real-world evidence generation. The benefits of OMOP include its scalability, flexibility, interoperability, support for advanced analytics, and the strong backing of a global, collaborative community.

Similarly, CDISC focuses on standardizing data for clinical trials to ensure regulatory compliance and facilitate submissions to authorities like the FDA and EMA. It is primarily utilized by pharmaceutical companies and clinical research organizations, using detailed models, such as SDTM and ADaM. In contrast, OMOP, developed by the OHDSI community, aims to harmonize observational healthcare data from sources like electronic health records and claims data, supporting large-scale observational research and real-world evidence studies. While CDISC is essential for pre-market approval processes, OMOP can also be used in post-market surveillance and comparative effectiveness research, providing a flexible and scalable framework for integrating diverse healthcare data.

### Limitations of our model

The RD-CDM model is a prototype model developed using the data elements of four domains of RDs; endocrinology, gastroenterology, pneumonology, and hematology. Therefore, the RD-CDM tables are limited to the focus domains. Although the RD-CDM modules cover most of the medical data, to be able to use it for other domains, a customization step might be necessary. Additionally, the genomic terminologies used in the RD-CDM are limited to the mutations and clinical elements that are part of data entities in our included cohorts. Although the genomic elements were mapped with a 100% success rate, we often faced two or more to one mappings [34]. Moreover, we used HPO terminologies for mapping the symptoms to OMOP, but the HPO terminology is still not integrated into the Athena terminology management tool for OMOP. Our implemented ETL only provides a quick and temporary solution. Further work is necessary to integrate HPO into OMOP terminology and introduce specific concept IDs for them.

### Lessons learned for the six individual customization steps

Bridging the gap between clinical experts and technical implementation is important for the design of such a model, which is why we consider the inclusion of experts from both domains and interdisciplinary collaboration as essential. Regular communication with the stakeholders helps to keep everyone aligned and informed about the progress and possible feedback. An iterative design process is essential to incorporate evolving requirements and insights.

1. A clear definition of the use case(s) must be provided.
  - This is of particular significance for interdisciplinary use cases, in which multiple domains are included (e.g., clinical, computational, organizational).
2. An interdisciplinary team of stakeholders should be defined based on the use case(s) as early as possible.
3. A list of diagnostic entities should be created together with the stakeholders for the targeted use case(s). A large group of medical experts is necessary for the definition and evaluation of data elements to ensure that the list of included elements in the final model is comprehensive. A consensus method for final models should be defined beforehand to objectively quantify the time period.
4. The use of use case-specific entities should be mapped to the modules of customizable RD-CDM.
5. For the Person, Diagnosis, Laboratory Findings, Procedure, and Medication, the FHIR-to-OMOP ETL process should be used to transfer the data into OMOP. Testing of the ETL processes using smaller synthetic data that has the same attributes as the real-world data is recommended to become accustomed to the logs and outcomes of the ETL process, especially if the real-world data is not directly available. For Genotypes, the direct ETL processes from CSV to OMOP CDM should be used to transfer the data into OMOP. The standard genomic vocabulary in OMOP has been used to map the mutations to OMOP. By writing the study-specific HPO concepts to the SOURCE_TO_CONCEPT_MAP table, a temporary solution has been provided for the integration of phenotype information into OMOP.
6. A robust ETL process is essential for the accurate transfer of data into the OMOP framework. This requires careful planning, thorough testing, and validation to process multiple data sources and maintain data integrity. Familiarity with the data and ETL tools is also key to effective implementation and problem solving.

### Outlook

This RD-CDM is the basis for the development of a decision support system, namely the SATURN platform, to be used at the point of care by the family practitioners. The platform will be equipped with different rule-based, case-based reasoning, and machine learning algorithms that aim to combine the available medical knowledge and clinical guidelines in the field with retrospective patient outcomes to support the diagnosis process of upcoming patients. General practitioners are often challenged with patients with symptoms that they do not have experience with. Therefore, this platform could support them in reaching a diagnosis in a shorter time.

A forward-looking usage for the platform could be integrating patient engagement features, such as a mobile app for tracking symptoms and facilitating communication with healthcare providers. This enhancement has the potential to empower patients significantly and enhance the management of their conditions. These additions could substantially boost the platform's effectiveness and reinforce its patient-centric orientation.

### Impact to RD

The complexity attached to RDs is due to their heterogeneity and geographical dispersion limiting available knowledge [2]. Patients are scattered in different countries and continents, and comprehensive data assembly is complicated not only by organizational, logistical, and communicative reasons but also by a lack of data collection standards and common frameworks. We provide a framework to easily integrate genetic information in large scale, multi-center studies, which in turn could reduce the amount of time spent on the characterization of phenotypes.

The customized RD-CDM based on OMOP can facilitate collaborations and investigations on an international level and in the long run improve patients’ quality of life through a faster diagnostic process.

## Conclusion

We refined our initially developed process for customized RD-CDM based on a prominent common data model in healthcare, OMOP CDM, using the data exchange standard FHIR [18]. The OMOP-based customized RD-CDM can enhance the harmonization of patient data in a standardized format that ensures international syntactic and semantic interoperability. That, in turn, enables—in addition to use the RD-CDM for decision support systems—retrospective studies including patient-level predictions possible using the tools offered by the OHDSI community and any other AI-based methods. Additionally, it allows attendance in international studies to deepen the findings by performing longitudinal studies on multi-center datasets.

### Supplementary Information

Additional file 1

## Data Availability

The ETL processes used to transfer data in this study including their mappings are available on our GitHub page: https://github.com/NajiaAhmadi/ETL-Genotype-Phenotype-to-OMOP. Additionally, we have generated a synthetic version of the hematology dataset used in this study as part of our previous publication [62] and have transformed it to the OMOP format as part another upcoming publication accepted in Medical Informatics Europe Conference 2024 (Hahn et al. 2024, Synthetic Data Generation in Hematology—Paving the Way for OMOP and FHIR Integration), which can be used for testing our pipeline. The synthetic data can be accessed from (https://github.com/NajiaAhmadi/AML-Synthetic-data-OMOP-version).

## Abbreviations

RDs: Rare diseases
CDM: Common data model
RD-CDM: Rare diseases common data model
OMOP: Observational medical outcomes partnership
OHDSI: Observational health data sciences and informatics
ETL: Extract, transform, load

## References

[1] Commissioner O of the. FDA. FDA; 2022 [cited 2023 Nov 27]. Rare Diseases at FDA. Available from: https://www.fda.gov/patients/rare-diseases-fda

[2] Wakap S, Lambert D, Olry A, Rodwell C, Gueydan C, Valérie L, et al. Estimating cumulative point prevalence of rare diseases: analysis of the Orphanet database. Eur J Hum Genet. 2019;16:28. 10.1038/s41431-019-0508-0 PMC6974615 31527858

[3] Aymé S, Schmidtke J. Networking for rare diseases: a necessity for Europe. Bundesgesundheitsblatt Gesundheitsforschung Gesundheitsschutz. 2007;50(12):1477–83. 18026888 10.1007/s00103-007-0381-9

[4] Bick D, Jones M, Taylor SL, Taft RJ, Belmont J. Case for genome sequencing in infants and children with rare, undiagnosed or genetic diseases. J Med Genet. 2019;56(12):783–91. 31023718 10.1136/jmedgenet-2019-106111 PMC6929710

[5] Wright CF, FitzPatrick DR, Firth HV. Paediatric genomics: diagnosing rare disease in children. Nat Rev Genet. 2018;19(5):253–68. 29398702 10.1038/nrg.2017.116

[6] Chu SY, Weng CY. Introduction to genetic/rare disease and the application of genetic counseling. Hu Li Za Zhi. 2017;64(5):11–7. 28948586 10.6224/JN.000063

[7] Stoller JK. The challenge of rare diseases. Chest. 2018;153(6):1309–14. 29325986 10.1016/j.chest.2017.12.018

[8] Tambuyzer E, Vandendriessche B, Austin CP, Brooks PJ, Larsson K, Miller Needleman KI, et al. Therapies for rare diseases: therapeutic modalities, progress and challenges ahead. Nat Rev Drug Discov. 2020;19(2):93–111. 31836861 10.1038/s41573-019-0049-9

[9] Mitani AA, Haneuse S. Small data challenges of studying rare diseases. JAMA Netw Open. 2020;3(3):e201965. 10.1001/jamanetworkopen.2020.1965. 32202640 10.1001/jamanetworkopen.2020.1965

[10] Shu L, Maroilley T, Tarailo-Graovac M. The Power of Clinical Diagnosis for Deciphering Complex Genetic Mechanisms in Rare Diseases. Genes. 2023;14(1):196. 36672937 10.3390/genes14010196 PMC9858967

[11] Personalised medicine [Internet]. 2023 [cited 2023 Nov 27]. Available from: https://research-and-innovation.ec.europa.eu/research-area/health/personalised-medicine_en

[12] Wilkinson MD, Dumontier M, Aalbersberg IJJ, Appleton G, Axton M, Baak A, et al. The FAIR Guiding Principles for scientific data management and stewardship. Sci Data. 2016;15(3): 160018. 10.1038/sdata.2016.18 PMC4792175 26978244

[13] dos Santos VB, Bernabé CH, Zhang S, Abaza H, Benis N, Cámara A, et al. Towards FAIRification of sensitive and fragmented rare disease patient data: challenges and solutions in European reference network registries. Orphanet J Rare Dis. 2022;17(1):436. 10.1186/s13023-022-02558-5. 36517834 10.1186/s13023-022-02558-5 PMC9749345

[14] Hageman IC, van Rooij IALM, de Blaauw I, Trajanovska M, King SK. A systematic overview of rare disease patient registries: challenges in design, quality management, and maintenance. Orphanet J Rare Dis. 2023;18(1):106. 10.1186/s13023-023-02719-0. 37147718 10.1186/s13023-023-02719-0 PMC10163740

[15] Torab-Miandoab A, Samad-Soltani T, Jodati A, Rezaei-Hachesu P. Interoperability of heterogeneous health information systems: a systematic literature review. BMC Med Inform Decis Mak. 2023;23:18. 36694161 10.1186/s12911-023-02115-5 PMC9875417

[16] A review of interoperability standards in E-health and imperatives for their adoption in Africa. S Afr Comput J. 2013;50. https://sacj.cs.uct.ac.za/index.php/sacj/article/view/176

[17] Olaronke I, Soriyan A, Gambo I, Olaleke J. Interoperability in healthcare: benefits, challenges and resolutions. Int J Innov Appl Stud. 2013;1(3):2028–9324.

[18] Overview—FHIR v5.0.0. [cited 2023 Nov 27]. Available from: https://www.hl7.org/fhir/overview.html

[19] The Medical Informatics Initiative’s core data set | Medical Informatics Initiative [Internet]. [cited 2023 Nov 27]. Available from: https://www.medizininformatik-initiative.de/en/medical-informatics-initiatives-core-data-set

[20] Kaliyaperumal R, Wilkinson MD, Moreno PA, Benis N, Cornet R, dos SantosVieira B, et al. Semantic modelling of common data elements for rare disease registries, and a prototype workflow for their deployment over registry data. J Biomed Semant. 2022;13(1):9. 10.1186/s13326-022-00264-6. 10.1186/s13326-022-00264-6 PMC8922780 35292119

[21] European Platform on Rare Disease Registration. [cited 2023 Nov 27]. Available from: https://eu-rd-platform.jrc.ec.europa.eu

[22] Choquet R, Maaroufi M, de Carrara A, Messiaen C, Luigi E, Landais P. A methodology for a minimum data set for rare diseases to support national centers of excellence for healthcare and research. J Am Med Inf Assoc. 2015;22(1):76–85. 10.1136/amiajnl-2014-002794. 10.1136/amiajnl-2014-002794 PMC4433369 25038198

[23] Bernardi FA, Mello de Oliveira B, Bettiol Yamada D, Artifon M, Schmidt AM, Machado Scheibe V, et al. The minimum data set for rare diseases: systematic review. J Med Internet Res. 2023;25:e44641. 37498666 10.2196/44641 PMC10415943

[24] Abaza H, Kadioglu D, Martin S, Papadopoulou A, Dos Santos VB, Schaefer F, et al. Domain-specific common data elements for rare disease registration: conceptual approach of a european joint initiative toward semantic interoperability in rare disease research. JMIR Med Inform. 2022;10(5): e32158. 35594066 10.2196/32158 PMC9166638

[25] Mullin AP, Corey D, Turner EC, Liwski R, Olson D, Burton J, et al. Standardized data structures in rare diseases: CDISC user guides for duchenne muscular dystrophy and Huntington’s disease. Clin Transl Sci. 2021;14(1):214–21. 32702147 10.1111/cts.12845 PMC7877853

[26] Kim HH, Park YR, Lee S, Kim JH. Composite CDE: modeling composite relationships between common data elements for representing complex clinical data. BMC Med Inform Decis Mak. 2020;20(1):147. 32620117 10.1186/s12911-020-01168-0 PMC7333279

[27] Maier C, Lang L, Storf H, Vormstein P, Bieber R, Bernarding J, et al. Towards implementation of OMOP in a German University Hospital Consortium. Appl Clin Inform. 2018;09(01):054–61. 10.1055/s-0037-1617452. 10.1055/s-0037-1617452 PMC5801887 29365340

[28] Ahmadi N, Peng Y, Wolfien M, Zoch M, Sedlmayr M. OMOP CDM can facilitate data-driven studies for cancer prediction: a systematic review. Int J Mol Sci. 2022;23(19):11834. 36233137 10.3390/ijms231911834 PMC9569469

[29] Ahmadi N, Peng Y, Wolfien M, Zoch M, Sedlmayr M. Cancer prediction on OMOP CDM—a rapid review. In German Medical Science GMS Publishing House;2022. p. DocAbstr. 23. 10.3390/ijms231911834 PMC9569469 36233137

[30] Ahmadi N, Zoch M, Sedlmayr B, Schuler K, Hahn W, Sedlmayr M, et al. Context-sensitive common data models for genetic rare diseases—a concept. In: Healthcare transformation with informatics and artificial intelligence. IOS Press; 2023 [cited 2023 Oct 23]. p. 139–40. 10.3233/SHTI230443 10.3233/SHTI230443 37386977

[31] Wagholikar KB, Dessai P, Sanz J, Mendis ME, Bell DS, Murphy SN. Implementation of informatics for integrating biology and the bedside (i2b2) platform as Docker containers. BMC Med Inf Decis Mak. 2018;18(1):66. 10.1186/s12911-018-0646-2. 10.1186/s12911-018-0646-2 PMC6048900 30012140

[32] Wolfien M, Ahmadi N, Fitzer K, Grummt S, Heine KL, Jung IC, et al. Ten topics to get started in medical informatics research. J Med Internet Res. 2023;25(1):e45948. 37486754 10.2196/45948 PMC10407648

[33] Zoch M, Gierschner C, Peng Y, Gruhl M, Leutner LA, Sedlmayr M, et al. Adaption of the OMOP CDM for rare diseases. Stud Health Technol Inform. 2021;27(281):138–42. 10.3233/SHTI210136 34042721

[34] Genomic Data Harmonization through the OMOP Standardized Vocabularies—OHDSI. [cited 2023 Nov 27]. Available from: https://www.ohdsi.org/2020-global-symposium-showcase-13/

[35] Buy M, Digan W, Chen X, Husson J, Ménager M, Rieux-Laucat F, et al. A multi-omics common data model for primary immunodeficiencies. Stud Health Technol Inform. 2022;6(290):56–60. 10.3233/SHTI220031 35672970

[36] Robinson PN, Köhler S, Bauer S, Seelow D, Horn D, Mundlos S. The human phenotype ontology: a tool for annotating and analyzing human hereditary disease. Am J Hum Genet. 2008;83(5):610–5. 18950739 10.1016/j.ajhg.2008.09.017 PMC2668030

[37] BfArM—ORPHAcodes [Internet]. [cited 2023 Aug 7]. Available from: https://www.bfarm.de/DE/Kodiersysteme/Kooperationen-und-Projekte/Orphanet/Orphanet-International/Orphacodes/_node.html

[38] Zhang XA, Yates A, Vasilevsky N, Gourdine JP, Callahan TJ, Carmody LC, et al. Semantic integration of clinical laboratory tests from electronic health records for deep phenotyping and biomarker discovery. Digit Med. 2019;2(1):1–9. 10.1038/s41746-019-0110-4 PMC6527418 31119199

[39] Reese JT, Blau H, Casiraghi E, Bergquist T, Loomba JJ, Callahan TJ, et al. Generalisable long COVID subtypes: findings from the NIH N3C and RECOVER programmes. EBioMedicine. 2023;87: 104413. 36563487 10.1016/j.ebiom.2022.104413 PMC9769411

[40] Ahmadi N, Zoch M, Kelbert P, Noll R, Schaaf J, Wolfien M, et al. Methods used in the development of common data models for health data: scoping review. JMIR Med Inf. 2023;11(1):e45116. 10.2196/45116 PMC10436118 37535410

[41] SATURN Projekt. [cited 2023 Nov 27]. Available from: https://www.saturn-projekt.de/

[42] Chen PPS. The entity-relationship model—toward a unified view of data. ACM Trans Database Syst. 1976;1(1):9–36. 10.1145/320434.320440. 10.1145/320434.320440

[43] fhir-to-omop. Observational Health Data Sciences and Informatics; 2023 [cited 2023 Nov 27]. Available from: https://github.com/OHDSI/ETL-German-FHIR-Core

[44] Informatics OHDS and Chapter 13 Patient-Level Prediction | The Book of OHDSI [Internet]. [cited 2023 Nov 27]. Available from: https://ohdsi.github.io/TheBookOfOhdsi/

[45] HADES. [cited 2023 Aug 4]. Available from: https://ohdsi.github.io/Hades/

[46] Ahmadi N. NajiaAhmadi/ETL-genotype-phenotype-to-OMOP: extract transform load (ETL) processes to write gene mutation data and phenotype data to OMOP CDM. [cited 2023 Nov 27]. Available from: https://github.com/NajiaAhmadi/ETL-Genotype-Phenotype-to-OMOP

[47] Hitachi Vantara Lumada and Pentaho Documentation. 2020 [cited 2023 Nov 27]. Pentaho Data Integration. Available from: https://help.hitachivantara.com/Documentation/Pentaho/Data_Integration_and_Analytics/9.1/Products/Pentaho_Data_Integration

[48] VORBEHALTEN IUAR. Orphanet: Suche /Krankheit. [cited 2023 Nov 27]. Available from: https://www.orpha.net/consor/cgi-bin/Disease_Search_Simple.php?lng=DE

[49] Athena. [cited 2023 Nov 27]. Available from: https://athena.ohdsi.org/search-terms/start

[50] BfArM—OPS. [cited 2023 Nov 27]. Available from: https://www.bfarm.de/EN/Code-systems/Classifications/OPS-ICHI/OPS/_node.html

[51] BfArM—LOINC. [cited 2023 Nov 27]. Available from: https://www.bfarm.de/EN/Code-systems/Terminologies/LOINC-UCUM/LOINC-and-RELMA/_node.html

[52] RxNorm Overview. U.S. National Library of Medicine; [cited 2023 Nov 27]. https://www.nlm.nih.gov/research/umls/rxnorm/overview.html

[53] Anatomical Therapeutic Chemical (ATC) Classification. [cited 2023 Nov 27]. https://www.who.int/tools/atc-ddd-toolkit/atc-classification

[54] Diagnosis-related group (DRG). [cited 2023 Nov 27]. https://www.ohdsi.org/web/wiki/doku.php?id=documentation:vocabulary:drg

[55] Schee Genannt Halfmann S, Mählmann L, Leyens L, Reumann M, Brand A. Personalized medicine: What’s in it for rare diseases? Adv Exp Med Biol. 2017;1031:387–404. 29214584 10.1007/978-3-319-67144-4_22

[56] Horgan D, Jansen M, Leyens L, Lal JA, Sudbrak R, Hackenitz E, et al. An index of barriers for the implementation of personalised medicine and pharmacogenomics in Europe. PHG. 2014;17(5–6):287–98. 10.1159/000368034 25401385

[57] Raycheva R, Kostadinov K, Mitova E, Bogoeva N, Iskrov G, Stefanov G, et al. Challenges in mapping European rare disease databases, relevant for ML-based screening technologies in terms of organizational, FAIR and legal principles: scoping review. Front Public Health. 2023;11:1214766. 37780450 10.3389/fpubh.2023.1214766 PMC10540868

[58] Sequence Variant Nomenclature [Internet]. [cited 2023 Nov 27]. Available from: https://varnomen.hgvs.org/

[59] Döhner H, Wei AH, Appelbaum FR, Craddock C, DiNardo CD, Dombret H, et al. Diagnosis and management of AML in adults: 2022 recommendations from an international expert panel on behalf of the ELN. Blood. 2022;140(12):1345–77. 10.1182/blood.2022016867. 35797463 10.1182/blood.2022016867

[60] Asche CV, Seal B, Kahler KH, Oehrlein EM, Baumgartner MG. Evaluation of healthcare interventions and big data: review of associated data issues. Pharmacoeconomics. 2017;35(8):759–65. 10.1007/s40273-017-0513-5. 28474299 10.1007/s40273-017-0513-5

[61] Kent S, Burn E, Dawoud D, Jonsson P, Østby JT, Hughes N, et al. Common problems, common data model solutions: evidence generation for health technology assessment. Pharmacoeconomics. 2021;39(3):275–85. 33336320 10.1007/s40273-020-00981-9 PMC7746423

[62] Eckardt JN, Hahn W, Röllig C, Stasik S, Platzbecker U, Müller-Tidow C, et al. Mimicking clinical trials with synthetic acute myeloid leukemia patients using generative artificial intelligence. npj Digit Med. 2024;7(1):1–11. 38509224 10.1038/s41746-024-01076-x PMC10954666

